# Acupuncture for Primary Osteoporosis: Evidence, Potential Treatment Prescriptions, and Mechanisms

**DOI:** 10.1155/2019/2705263

**Published:** 2019-06-12

**Authors:** Haixiong Lin, Xiaotong Wang, Yingjie Mo, Chunni Lin, Nenggui Xu, Feng Huang, Yongjun Chen

**Affiliations:** ^1^The First School of Clinical Medicine, Guangzhou University of Chinese Medicine, Guangzhou 510405, China; ^2^South China Research Center for Acupuncture and Moxibustion, Medical College of Acu-Moxi and Rehabilitation, Guangzhou University of Chinese Medicine, Guangzhou 510006, China; ^3^Dongguan Hospital of Traditional Chinese Medicine, Dongguan 523127, China; ^4^School of Foreign Languages, Xinhua College of Sun Yat-sen University, Dongguan 523133, China; ^5^The First Affiliated Hospital of Guangzhou University of Chinese Medicine, Guangzhou 510405, China

## Abstract

Many clinical trials and meta-analyses related to acupuncture for osteoporosis (OP) have been published. However, identifying the evidence from these studies still remains a challenge for acupuncturists. We conducted a systematic search of the Chinese Biomedical Medicine (CBM), VIP Database, Wanfang Data, China National Knowledge Infrastructure (CNKI), PubMed, Springer, Cochrane Library, and Embase to identify relevant trials, systematic reviews, and/or meta-analyses up to October 31, 2018. Data were extracted to assess the methodological quality using Veritas plots and to explore potential acupuncture prescriptions using the Traditional Chinese Medicine inheritance support system (TCMISS). In addition, potential mechanisms of core acupoints identified by data mining were summarized based on published studies. A total of 218 clinical trials and ten meta-analyses were included, involving 212 acupuncture prescriptions, 102 acupoints, 13 meridians, three extra meridians, and one Ashi point. The mean Veritas score of publication year, type of study, Assessment of Multiple Systematic Reviews 2, Preferred Reporting Items for Systematic Reviews and Meta-Analyses, heterogeneity, and publication bias were 5.5, 7.2, 6, 5.6, 5.8, and 7.5, respectively. The study of Pan et al. received the highest Veritas score of 8.67 points. The most frequently used meridian was BL. Acupoint combinations BL23 and BL20, BL23 and GV4, and BL23 and ST36 were used frequently. The core acupoints association networks were acupoints BL23, BL20, ST36, GV4, SP6, CV4, and KI3. The potential mechanisms of core acupoints involved upregulated expression of members in OPG/RANKL, Wnt/*β*-catenin, and MAPK pathways, such as LRP5, *β*-catenin, Runx2, and OPG. In conclusion, our Veritas plots enable acupuncturists to evaluate key attributes of meta-analysis quality related to acupuncture for primary OP and to improve the quality of evidence-based medicine relating to acupuncture. Data mining analysis revealed an association network of meridians, acupoint combinations, core acupoints, and the underlying mechanisms of acupuncture for primary OP.

## 1. Introduction

Osteoporosis (OP) is a disease characterized by low bone mineral density (BMD) and a high risk of fractures [1], with clinical manifestations of pain, muscle weakness, and fractures [2]. Approximately 9 million people worldwide suffer from OP or fragility (low trauma) fractures each year [3]. Previous studies have found that acupuncture could significantly increase the BMD of lumbar vertebrae, the values of trabecular area, and trabecular bone number, as well as reducing trabecular separation in postmenopausal OP rats [4]. Similar but slightly different results occur simultaneously in patients with OP [5]. In recent years, the number of patients seeking complementary and alternative therapies, including acupuncture, has grown continuously in clinical practice [6], leading to a large number of clinical trials and meta-analyses related to acupuncture for OP being published in various journals and databases [5, 7]. However, these studies are influenced by a series of biases and increasingly include nonrandomized clinical trials [8]. Identifying quality evidence and potential treatment strategies from these studies and applying it to clinical practice still remains a challenge for clinicians [9, 10]. Therefore, identifying clinical evidence and exploring treatment strategies of acupuncture has become an urgent problem.

The Veritas plot is a graphic tool adapted from radar plots to describe multiattribute data [10]. This technique was first applied to identify the highest quality meta-analytical literature of cardiac surgery [10]. As an evidence-synthesis graphic tool, Veritas plots are now widely used to identify and interpret variability in meta-analyses and to help clinicians use the highest quality evidence of acupuncture in clinical practice [11]. However, Veritas plots have not been applied to acupuncture for primary OP. TCMISS is a data mining tool invented by the* Institute of Chinese Materia Medica China Academy of Chinese Medical Sciences* for exploring potential treatment strategies of acupuncture or Chinese medicine [12, 13]. It includes different functions such as text mining, association rules analysis, and complex system entropy methods, making it easy and convenient for researchers without algorithmic experience to use it for data mining [14]. It can be used to guide clinicians in selecting appropriate acupoints or Chinese herbs to treat specific diseases [14, 15]. However, data mining has not been conducted to explore potential acupuncture prescriptions for primary OP.

Given the uncertainty about the quality of meta-analyses and the treatment strategy of acupuncture for OP, we used Veritas plots to assess quality evidence of acupuncture and used TCMISS to explore potential treatment strategies of acupuncture for primary OP. In addition, we summarized the major antiosteoporotic effects and potential mechanisms of core acupoints identified by data mining. These will help clinicians establish a specific acupuncture prescription for OP and use it in clinical practice.

## 2. Methods

### 2.1. Participants

Patients with primary OP were included as participants in this study. The diagnostic criteria for primary OP were based on the 1999 World Health Organization criteria: BMD is measured by dual energy X-ray absorptiometry as a T-score below -2.5 standard deviations (SD) in the femur neck, lumbar spine, or total femur region [16]. There were no restrictions on age, sex, race, language, or region.

### 2.2. Selection Criteria

This study included randomized control trials (RCTs) and/or clinical control trials (CCTs) and systematic reviews and/or meta-analyses of acupuncture for OP. Cross-sectional studies, comments, cohort studies, animal experiments, and reviews were excluded.

### 2.3. Search Strategy

A systematic electronic search was conducted of CBM, VIP Database, Wanfang Data, CNKI, PubMed, Springer, Cochrane Library, and Embase for relevant RCTs and/or CCTs and systematic reviews and/or meta-analyses. All of the databases were searched from their inception to October 31, 2018. The search algorithm for PubMed was as follows: (“acupuncture” or “electroacupuncture” or “needle warming therapy” or “eye acupuncture” or “foot acupuncture” or “wrist-ankle acupuncture” or “hand acupuncture” or “hand-foot acupuncture” or “face acupuncture” or “nose acupuncture” or “Ashi points”) and (“age-related osteoporosis” or “senile osteoporosis” or “postmenopausal osteoporosis” or “primary osteoporosis”) and (“randomized controlled trial” or “trial” or “clinical trial” or “systematic review” or “meta-analysis”), with no restriction on subheadings. Similar but adapted search terms were used in other databases. Clinical trials published in abstract form were excluded unless sufficient information could be obtained from the abstract or authors. Reference lists of the retrieved papers were also reviewed to discover other studies, and studies not included in the databases mentioned previously were additionally searched.

### 2.4. Data Selection

The results from different databases were imported into NoteExpress 3.2.0 (http://www.inoteexpress.com/aegean/). Duplicate studies were then identified and deleted with NoteExpress 3.2.0. Two authors conducted data selection independently (Lin HX and Wang XT), reviewed the abstracts and full texts, and extracted the relevant information from the included studies. Any discrepancies were resolved by consensus.

### 2.5. Data Extraction

Two reviewers (Wang XT and Lin HX) extracted study information for each eligible study, including the first author names, publication year, data sources, time range for inclusion in the study, detailed search strategies, search process, sample sizes, number of patients, bias assessment tools, outcomes, and methods of reporting publication bias. Data was checked by Yingjie Mo.

### 2.6. Veritas Score Indicators

Veritas scores were determined for publication year, study type, Assessment of Multiple Systematic Reviews (AMSTAR) 2 score, Preferred Reporting Items for Systematic Reviews and Meta-Analyses (PRISMA) score, heterogeneity, and publication bias to assess the quality of included systematic reviews or meta-analyses. The year a study was published was an important factor of heterogeneity, as disease and acupuncture techniques may change over time, whereas technical expertise may have led to unfavorable results in early studies [17]. The AMSTAR 2, updated in 2017, could provide readers with a better summary assessment to determine whether the systematic review has met the methodological safeguards against bias [18]. PRISMA statement could accurately reflect the ability of researchers to write in a comprehensible manner, instead of the way they conducted the review [19, 20]. Heterogeneity in study design or intervention has a significant impact on the conduct and outcomes of a meta-analysis [21] and often affects acupuncturists in some important decisions, such as whether a treatment is applicable to all targeted people [22]. Publication bias is an inherent problem in meta-analyses because some studies are not published in indexed journals, and some negative results were not publicly available [23, 24].

### 2.7. Ranking of Evidence

The meta-analyses were ranked according to six categories: year of publication, study type, AMSTAR2 score, PRISMA score, heterogeneity, and publication bias. For publication year, if the publication year of multiple studies was the same, we ranked studies by the time range for inclusion in the study. In the AMSTAR 2 score, if the study meets the criteria of AMSTAR 2 item, it scores 1 point in this item; 0.5 points for those that partially satisfy the criteria; otherwise 0 points. In the PRISMA score, 2 points were given for studies that completely meet the criteria of the PRISMA item; 1 point for those that partially satisfy the criteria; otherwise 0 points. The heterogeneity score is the average of the heterogeneity scores for clinical outcomes. Heterogeneity among studies was calculated using the chi-square test and I^2^ statistic [25]. 0% ≤ I^2^ ≤ 50% indicated no heterogeneity and the score is 3 points, 50% < I^2^ ≤ 75% was considered slightly significant heterogeneity and scores 2 points, and I^2^ > 75% was regarded as significant heterogeneity and scores 1 point.

The scoring system was applied as follows [10]: in each category, the best study got the highest score of n, where n = the number of studies. The second best study got n-1 points, and so on. If two studies performed equally well, the included study with the next highest score would get n-2 points. We considered the Veritas score as a summary statistic. The Veritas score of each study is the average scores in the six dimensions of quality.

### 2.8. Data Mining Analysis

Data were analyzed by TCMISS Version 2.5 (*invented by Institute of Chinese Materia Medica China Academy of Chinese Medical Sciences*). We first analyzed the frequency of acupoints, meridians, and common acupoint combinations using text mining. Then we conducted an association rule analysis to determine the support value of different acupoints and the core acupoints association network of acupuncture for primary OP. We set the “the degree of support” to 60, which means the frequency of A and B appearing at the same time is 60. The “confidence level” was set to 0.6, which means that when A occurs, the probability of B appearing is 60%. We also further analyzed the cooccurrence matrix of the top 23 acupoints using Heml 1.0.3.7 (http://hemi.biocuckoo.org/down.php). The results were represented by a heat map.

### 2.9. Summary of Major Antiosteoporotic Effects and Potential Mechanisms of Core Acupoints

The major antiosteoporotic effects and underlying mechanisms of core acupoints identified by data mining were summarized using published studies, thereby providing evidence for the patients and acupuncturists of the applications of acupuncture for primary OP

## 3. Results

### 3.1. Eligible Studies

**Figure 1:**
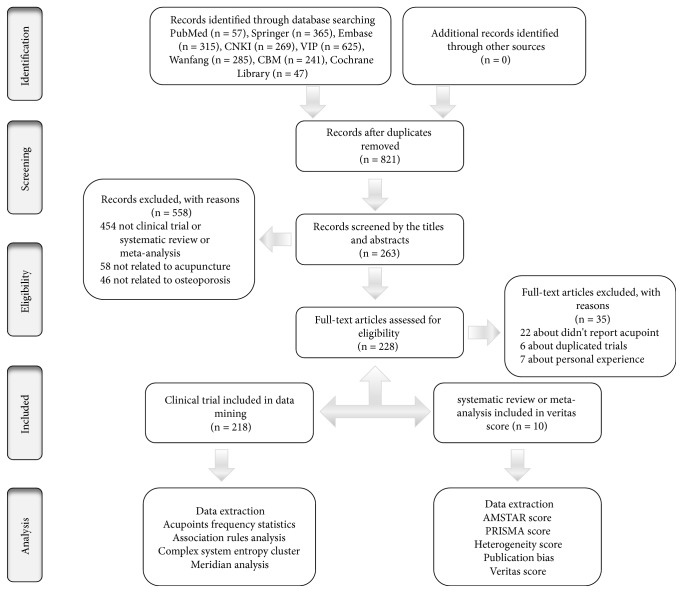
*Flow chart of the research process*. CBM = Chinese Biomedical Medicine, CNKI = China National Knowledge Infrastructure, AMSTAR = Assessment of Multiple Systematic Reviews tool, and PRISMA = Preferred Reporting Items for Systematic Reviews and Meta-Analyses.

The research process is summarized in Figure 1. We firstly identified 2204 citations through an initial search and identified 821 potential articles after removing duplicated records. After screening the titles and abstracts, 558 studies were deleted. We included 228 studies after screening the full text of these 558 studies. In total, 218 studies were clinical trials and the acupoint characteristics were analyzed using TCMISS Version 2.5. Ten studies were meta-analyses and the evidence was ranked using Veritas scores. The baseline characteristics of the meta-analyses are presented in Table 1. The meta-analyses were published between 2014 and 2018, with data from 12 databases including Embase, Medline, the Cochrane Library, ScienceDirect, PubMed, AMED, CBM, VIP Database, Wanfang Data, CNKI, Web of Science, and The Cochrane Central Register of Controlled Trials. The databases were searched from their inception to October 31, 2018, in the latest meta-analysis. A total of 138 clinical trials involving 11,232 patients were included in 10 meta-analyses. Five meta-analyses provided a detailed search strategy. Six meta-analyses had search processes that were performed independently by two reviewers. In the bias assessment, five meta-analyses used Cochrane Collaboration's risk of bias tool, while others used the Jadad scale. Four meta-analyses did not report publication bias.

### 3.2. Veritas Score

**Figure 2:**
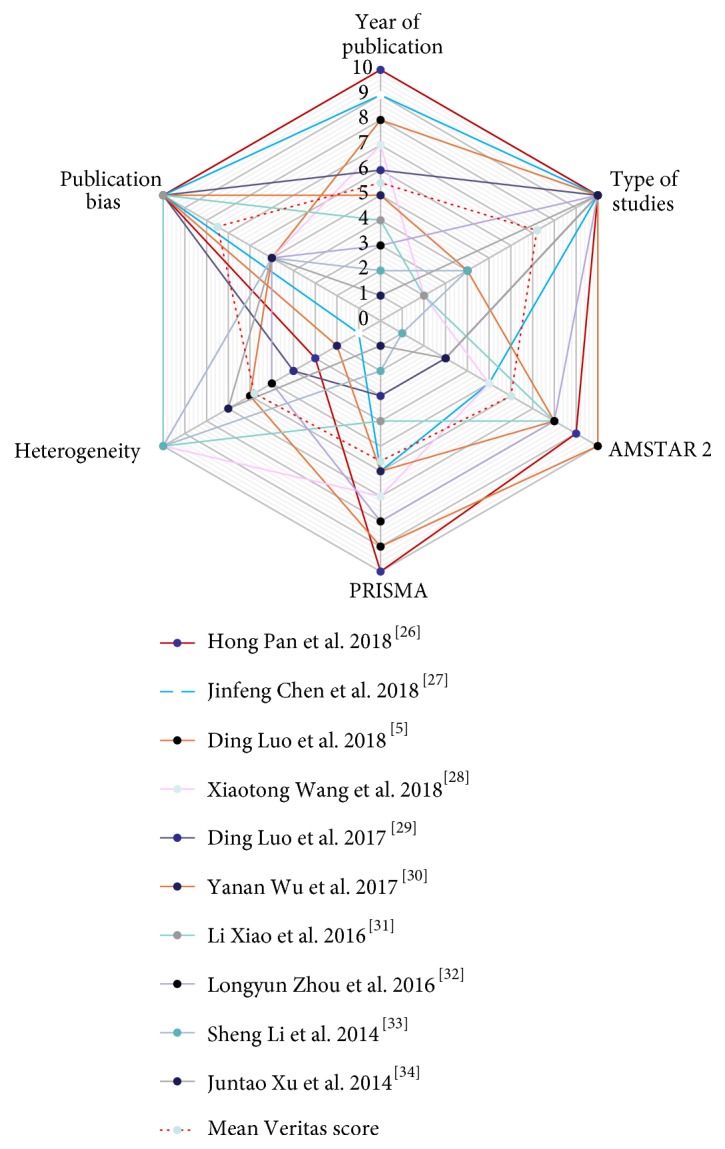
*Veritas plots for included studies*. AMSTAR = Assessment of Multiple Systematic Reviews tool; PRISMA = Preferred Reporting Items for Systematic Reviews and Meta-Analyses.

**Figure 3:**
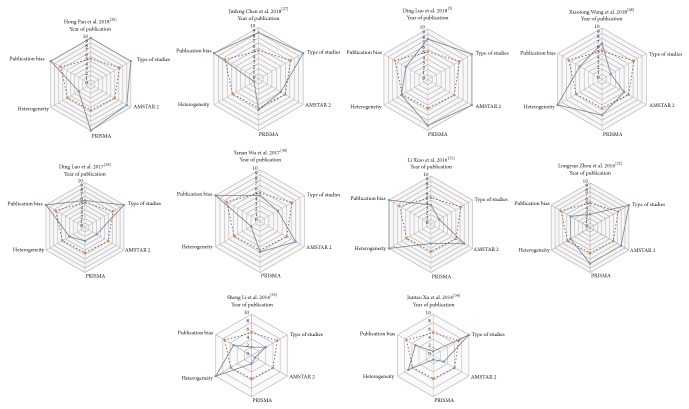
*Comparison of Veritas score in all included studies*. AMSTAR = Assessment of Multiple Systematic Reviews tool; PRISMA = Preferred Reporting Items for Systematic Reviews and Meta-Analyses.

The scores of included meta-analyses are presented in Table 2. The detailed heterogeneity score, AMSTAR 2 score, and PRISMA score of included studies are shown in Supplementary Tables 1, 2, and 3, respectively. We also developed summary Veritas plots of the 10 studies (Figure 2). The mean Veritas scores of publication year, type of study, AMSTAR 2 score, PRISMA score, heterogeneity, and publication bias, were 5.5, 7.2, 6, 5.6, 5.8, and 7.5, respectively. The number of meta-analyses that exceeded the mean Veritas scores of publication year, type of study, AMSTAR 2 score, PRISMA score, heterogeneity, and publication bias was 5, 6, 5, 6, 5, and 5, respectively. The study with the highest Veritas score was by Pan et al. (2018), with 8.67 points, while the lowest Veritas score was Li et al. (2014), with 4 points. There were 5 studies with Veritas scores ≥ 6.27 points. In Figure 3, the individual Veritas scores for all of the meta-analyses are plotted.

### 3.3. Frequency Statistics of Acupoints

A total of 212 acupuncture prescriptions were included, which involved 102 acupoints. The top 30 acupoints of acupuncture for OP are presented in Table 3. Acupoints BL23, BL20, ST36, and GV4 were used in more than 60% of the acupuncture prescriptions, with frequency of 178, 120, 112, and 111, respectively. There were 10 acupoints with frequency ≥ 50, which were BL23, BL20, ST36, GV4, SP6, CV4, KI3, GV3, BL40, and BL11.

### 3.4. Frequency Statistics of Meridians

Meridians and acupoints used in acupuncture therapy for primary OP are shown in Table 4. A total of 13 meridians, three extra meridians, and one Ashi point were used in acupuncture for primary OP. There were six meridians used over 100 times, including stomach meridian of foot-yangming (ST), spleen meridian of foot-taiyin (SP), governor vessel (GV), gallbladder meridian of foot-shaoyang (GB), conception vessel (CV), and bladder meridian of foot-taiyang (BL). The most frequently used meridian was BL, which was used 640 times and involved 31 acupoints. The second most commonly used meridian was GV, which was used 269 times and involved 13 acupoints. CV was the third most frequently used meridian, with 147 times and nine points.

### 3.5. Common Acupoint Combinations

**Figure 4:**
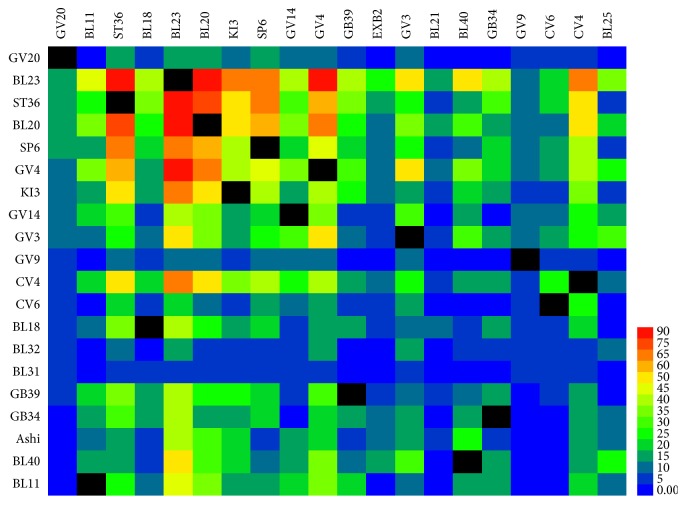
Cooccurrence matrix of acupoints.

The common acupoint combinations of acupuncture for primary OP are presented in Table 5. A total of 15 common acupoint combinations were frequently used over 60 times. There were three acupoint combinations with frequency ≥ 100: BL23 and BL20, BL23 and GV4, and BL23 and ST36. The acupoint combination BL23 and BL20 was used 117 times in acupuncture therapy for OP, accounting for about 50% of acupuncture prescriptions. The second most frequently used acupoint combination was BL23 and GV4, with 104 times. Acupoints BL20 combined with ST36 and BL23, BL20 combined with ST36 were used as many as 76 times. These results were consistent with the cooccurrence matrix of acupoints, which are shown in Figure 4. There were 18 pairs of acupoints with a cooccurrence frequency above 50, of which four pairs were above 75.

### 3.6. Association Rules of Acupoints

**Figure 5:**
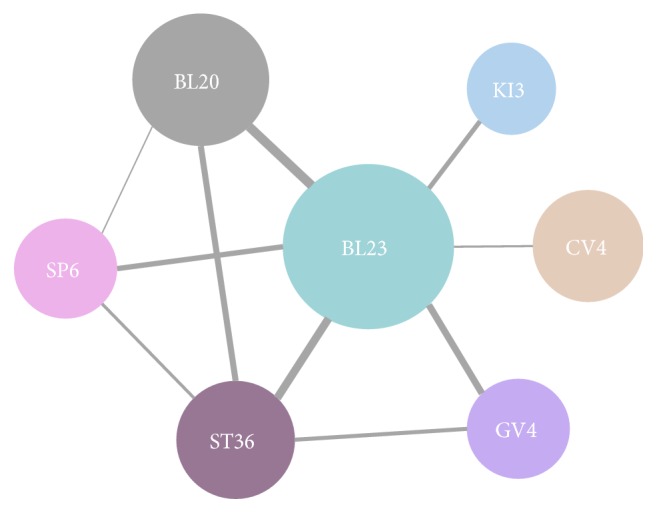
*Core acupoints association network of acupuncture for primary osteoporosis*. The size of the circle represents the frequency and the width of the line indicates the degree of support.

We present the association rules for acupoints in Table 6. There were 22 acupoint combinations with confidence levels ranging from 60% to 98.7%, seven of which were above 90%, such as acupoint BL20, ST36 -> BL23; BL20 -> BL23; CV4 -> BL23; BL20, GV4 -> BL23; SP6 -> BL23; GV4 -> BL23; KI3 -> BL23. The acupoint combinations of BL20 and ST36 with BL23 have the highest confidence level of 98.7%, which means that when BL20 and ST36 were selected, the probability of selecting BL23 was 98.7%. The acupoint combination of BL20 and BL23 has the second highest confidence level of 97.5%. The core acupoints association network of acupuncture for primary OP is shown in Figure 5, including acupoints BL23, BL20, ST36, GV4, SP6, CV4, and KI3. The degree of support from strong to weak was acupoint BL23 combined with BL20, BL23 with ST36, BL23 with GV4, BL20 with ST36, BL23 with SP6, BL23 with KI3, ST36 with GV4, ST36 with SP6, BL23 with CV4, and SP6 with BL20.

### 3.7. Beneficial Effects of Core Acupoints on Primary OP and Potential Mechanisms

Representative examples of major antiosteoporotic effects of core acupoints and potential mechanisms are presented in Table 7. The prevention of primary OP in acupoints BL23 and BL20 was mainly to reduce the loss of bone mass, increase femoral BMD, and improve the microstructure of bone tissue. The role of acupoints ST36, SP6, CV4, and KI3 in preventing OP was mainly to increase E_2_ levels and BMD. The antiosteoporotic effects of acupoints GV4 and CV4 were to reduce the loss of bone mass and increase the femoral BMD. The potential antiosteoporotic mechanisms in acupoints BL23, BL20, and GV4 were to upregulate the ratio of OPG/RANKL, and LRP5, *β*-catenin, and Runx2 expression. In addition, acupoints BL23 and BL20 also could downregulate the expression of phosphorylated (p)-p38 and p-JNK. The potential antiosteoporosis mechanism in acupoints SP6 and KI3 was to decrease the concentration of TNF*α* and IgM. Acupoint ST36 could reduce OPGL expression and upregulate the expression levels of RANKL mRNA. Acupoint CV4 could upregulate the expression levels of ER-*α* and ERA-*α* mRNA in uterus, hypothalamus, and spleen.

## 4. Discussion

In our study, we presented a novel application of Veritas plots that acupuncturists can use to evaluate key attributes of meta-analysis quality without extensive narratives. A total of 10 meta-analyses were included. The mean Veritas score of publication year, type of study, AMSTAR 2, PRISMA, heterogeneity, and publication bias were 5.5, 7.2, 6, 5.6, 5.8, and 7.5, respectively, indicating that the included meta-analyses performed well in the following areas: timely update results, high consistency of study type, compliance with PRISMA statements, and low heterogeneity. However, the statement of the review method, the assessment of the potential impact of risk of bias in individual studies on the results, the list of excluded studies, funding sources, and conflicts of interest in AMSTAR 2 were insufficient. Some meta-analyses did not assess publication bias, indicating that we should pay more attention to the methodological guarantee of bias, and consider the clinical value of the results carefully. A similar phenomenon also occurs in acupuncture treatment of anxiety disorders [41], which indicates a direction to further improve the quality of research. With the help of the Veritas scores, we ultimately found that the study of Pan et al. [26] performed well in the year of publication, type of study, AMSTAR 2 score, PRISMA score, and publication bias and received the highest Veritas score of 8.67 points.

In this study, we used TCMISS to investigate the meridians, acupoint combinations, and core acupoints for primary OP. A total of 212 acupuncture prescriptions were included, including 102 acupoints, 13 meridians, three extra meridians, and one Ashi point. The most frequently used meridian was BL, which was used 640 times and involved 31 acupoints. The second most frequently used meridian was GV. Experiments in ovariectomized rats have demonstrated that acupuncture at BL and GV acupoints could alleviate osteoporosis by regulating the OPG/RANKL and Wnt/beta-catenin signaling pathways [38]. Acupoint combination BL23 and BL20 was used in about 50% of acupuncture prescriptions, with a frequency of 117. The second most frequent acupoint combination was BL23 and GV4, and the third was BL23 and ST36. A clinical study has demonstrated that electroacupuncture stimulation at BL20/BL23 could increase levels of osteocalcin and the BMD of lumbar vertebrae, as well as improving bone microstructure in the femur [4]. The core acupoints association network of acupuncture for primary OP included BL23, BL20, ST36, GV4, SP6, CV4, and KI3. BL23 and KI3 [6] and BL23 and GV4 [42] have been demonstrated in randomized controlled trials to improve quality of life and reduce pain in patients with primary OP. At the same time, we summarized the major antiosteoporotic effects and potential mechanisms of core acupoints based on published studies. The major antiosteoporotic effects of core acupoints were increased BMD and reduced loss of bone mass. The underlying antiosteoporotic mechanisms were to upregulate the expression of members in OPG/RANKL, Wnt/*β*-catenin, and MAPK pathways, such as LRP5, *β*-catenin, Runx2, and OPG.

## 5. Limitations

There are some limitations in this study. First, we did not assess the impact of population factor on the results, which is different from the Panesar et al. study [10]. Second, the number of clinical trials, especially the multicenter randomized controlled clinical trials that validated the experience of well-known Chinese medicine practitioners, is small. This may limit the applications of the results. Third, the acupoint combinations and core acupoints of acupuncture for primary OP were rarely confirmed by large-scale multicenter clinical trials or animal experiments.

## 6. Conclusion

Our Veritas plots enable acupuncturists to evaluate key attributes of meta-analysis quality related to acupuncture for primary OP and to improve the quality of evidence-based medicine related to acupuncture. Data mining analysis revealed an associated network involving meridians, acupoint combinations, core acupoints, and the underlying mechanisms of acupuncture for primary OP. Future large-sample multicenter RCTs and animal experiments should be conducted to confirm our findings.

## Figures and Tables

**Table 1:** Baseline characteristics of studies included in our example.

Studies	Data Sources	Time	Detailed search strategy	Search process	Number of studies	Number of patients (E/C)	Bias assessment tool	Outcomes	Report publication bias
Hong Pan et al. 2018 [26]	Embase, Medline, the Cochrane library, ScicenceDirect, PubMed, AMED, CBM, Wanfang, CNKI, VIP	Before April 2017	Yes	Two reviewers independently	35	1517/1465^*∗*^	Cochrane Collaboration's risk of bias tool	clinical therapeutic effect, BMD, Pain VAS, Serum Calcium, Serum Alkaline Phosphatase, E2	Funnel plot

Jinfeng Chen et al. 2018 [27]	PubMed, CNKI, CBM, VIP, Wanfang	Before November 2016	Yes	Not mention	12	515/481	Jadad Scale	clinical therapeutic effect, BMD, Pain scale, BGP, Serum Alkaline Phosphatase, safety	Funnel plot

Ding Luo et al. 2018 [5]	Medline, Embase, Cochrane Library, Web of Science, CNKI, the Chinese VIP database and Wangfang database	Before June 30, 2016	Yes	Two reviewers independently	9	303/269^*∗*^	Cochrane Collaboration's risk of bias tool	BMD of lumbar vertebrae, BMD of femoral neck, Ward's triangle and greater Trochanter, pain VAS	None

Xiaotong Wang et al. 2018 [28]	CNKI, Wanfang, CBM, VIP, PubMed	Before June 1, 2016	Yes	Two reviewers independently	4	93/93	Cochrane Collaboration's risk of bias tool	clinical therapeutic effect, BMD, E2, BGP, Ca/Cr	None

Ding Luo et al. 2017 [29]	CNKI, Wanfang, VIP, PubMed, Embase, The Cochrane Central Register of Controlled Trials	Before October 1, 2016	None	Not mention	23	959/917^*∗*^	Cochrane Collaboration's risk of bias tool	clinical therapeutic effect, Pain scale, BMD of lumbar vertebra, E2, testosterone, BGP, TCM symptom score	Funnel plot, Egger's test

Yanan Wu et al. 2017 [30]	CNKI, Wanfang, VIP, PubMed, EMBASE, The Cochrane Central Register of Controlled Trials	Before December 1, 2015	None	Two reviewers independently	11	487/485	Cochrane Collaboration's risk of bias tool	Pain scale, BMD of lumbar vertebra, TCM symptom score, effectiveness	Funnel plot, Egger's test

Li Xiao et al. 2016 [31]	CNKI, VIP, Wanfang, CBM, PubMed, Cochrane Central Register of Controlled Trials (CENTRAL), Embase	2006.1-2016.7	None	Two reviewers independently	10	477/476^*∗*^	Jadad Scale	clinical therapeutic effect, E2, BMD	Funnel plot

Longyun Zhou et al. 2016 [32]	CNKI, VIP, CBM, Wanfang, Chinese Clinical Trial Registry, PubMed, Cochrane Library, Web of Science, Embase, AMED	Before September 1, 2015	Yes	Two reviewers independently	16	658/606	Jadad Scale	clinical therapeutic effect, Pain measurement, BMD, safety	Funnel plot

Sheng Li et al. 2014 [33]	CNKI, VIP, Wanfang, CBM, and Luzhou Medical College Library	Before October 1, 2013	None	Not mention	9	365/322	Jadad Scale	clinical therapeutic effect, BMD, E2	None

Juntao Xu et al. 2014 [34]	CNKI, VIP, Wanfang	2003-2011	None	Not mention	9	373/371	Jadad Scale	clinical therapeutic effect, bone pain, BMD of lumbar vertebra	None

*Note*. RCT, randomized control trials; CCT, clinical control trial; BMD, Bone Mineral Density; BGP, bone Gla protein; VAS, visual analog scale; E2, estradiol; TCM, traditional Chinese Medicine; E, experimental group; C, control group; CBM, Chinese Biomedical Medicine; CNKI, China National Knowledge Infrastructure.

*∗*The data is erroneous in the included studies. We have corrected it.

**Table 2:** Scores of included studies.

Studies	Studies characteristics (score)												Veritas score
	Year of publication		Type of studies		AMSTAR 2 score		PRISMA score		Heterogeneity		Publication bias		
Hong Pan et al. 2018 [26]	2018	(10)	RCT	(10)	11	(9)	47	(10)	1.64	(3)	Partially assessed	(10)	8.67

Jinfeng Chen et al. 2018 [27]	2018	(9)	RCT	(10)	9	(5)	41	(6)	1.4	(1)	Partially assessed	(10)	6.83

Ding Luo et al. 2018 [5]	2018	(8)	RCT	(10)	11.5	(10)	46	(9)	2.2	(6)	None	(5)	8

Xiaotong Wang et al. 2018 [28]	2018	(7)	RCT or CCT	(2)	9	(5)	42	(7)	3	(10)	None	(5)	6

Ding Luo et al. 2017 [29]	2017	(6)	RCT	(10)	8	(3)	36	(3)	1.75	(4)	Partially assessed	(10)	6

Yanan Wu et al. 2017 [30]	2017	(5)	RCT or quasi-RCT	(4)	9.5	(8)	41	(6)	1.5	(2)	Partially assessed	(10)	5.83

Li Xiao et al. 2016 [31]	2016	(4)	RCT or CCT	(2)	9.5	(8)	39	(4)	3	(10)	Partially assessed	(10)	6.33

Longyun Zhou et al. 2016 [32]	2016	(3)	RCT	(10)	9.5	(8)	44	(8)	2	(5)	None	(5)	6.5

Sheng Li et al. 2014 [33]	2014	(2)	RCT or quasi-RCT	(4)	7.5	(1)	35	(2)	3	(10)	None	(5)	4

Juntao Xu et al. 2014 [34]	2014	(1)	RCT	(10)	8	(3)	25	(1)	2.33	(7)	None	(5)	4.5

Mean Veritas score	5.5		7.2		6		5.6		5.8		7.5		6.27

*Note*. AMSTAR = Assessment of Multiple Systematic Reviews tool; PRISMA = Preferred Reporting Items for Systematic Reviews and Meta-Analyses.

**Table 3:** Top 30 acupoints of acupuncture for primary osteoporosis.

Number	acupoints	Frequency	Number	acupoints	Frequency	Number	acupoints	Frequency
1	BL23	178	11	GV14	49	21	SP10	19
2	BL20	120	12	GB39	49	22	CV12	19
3	ST36	112	13	GB34	46	23	BL17	19
4	GV4	111	14	Ashi	46	24	BL21	18
5	SP6	77	15	BL18	44	25	BL52	17
6	CV4	77	16	BL25	41	26	BL32	16
7	KI3	76	17	EX-B2	37	27	BL54	16
8	GV3	60	18	CV6	31	28	GV9	12
9	BL40	59	19	GB30	21	29	CV8	12
10	BL11	53	20	GV20	20	30	BL26	11

**Table 4:** Meridians and acupoints used in acupuncture therapy for primary osteoporosis.

Meridian	Times	Number	Acupoints
Bladder meridian of foot-taiyang (BL)	640	31	BL11(53), BL12(2), BL13(3), BL14(1), BL15(1), BL16(1), BL17(19), BL18(44), BL19(1), BL20(120), BL21(18), BL22(1), BL23(178), BL24(7), BL25(41), BL26(11), BL27(1), BL28(1), BL31(8), BL32(16), BL33(1), BL34(1), BL36(9), BL37(1), BL40(59), BL43(1), BL52(17), BL54(16), BL57(5), BL60(2), BL67(1)
Governor vessel (GV)	269	13	GV1(1), GV2(3), GV3(60), GV4(111), GV5(2), GV6(2), GV8(1), GV9(12), GV11(2), GV12(2), GV14(49), GV16(4), GV20(20)
Conception vessel (CV)	147	9	CV4(77), CV6(31), CV8(12), CV9(2), CV10(3), CV11(1), CV12(19), CV13(1), CV17(1)
Gallbladder meridian of foot-shaoyang (GB)	135	7	GB20(5), GB21(3), GB30(21), GB31(10), GB34(46), GB35(1), GB39(49)
Stomach meridian of foot-yangming (ST)	131	6	ST24(4), ST25(6), ST26(3), ST31(2), ST34(4), ST36(112)
Spleen meridian of foot-taiyin (SP)	114	5	SP3(5), SP6(77), SP9(7), SP10(19), SP15(6)
Kidney meridian of foot-shaoyin (KI)	89	8	KI1(2), KI3(76), KI4(2), KI7(2), KI10(3), KI13(2), KI14(1), KI16(1)
Ashi point	46	1	Ashi(46)
Points of back (EX-B)	40	2	EX-B2(37), EX-B5(3)
Liver meridian of foot-jueyin (LR)	10	3	LR3(8), LR8(1), LR14(1)
Large intestine meridian of hand-yangming (LI)	10	4	LI4(4), LI11(3), LI13(1), LI15(2)
Lung meridian of hand-taiyin (LU)	6	3	LU5(3), LU7(2), LU9(1)
Small intestine meridian of hand-taiyang (SI)	5	4	SI3(1), SI9(1), SI11(2), SI15(1)
Pericardium meridian of hand-jueyin (PC)	4	1	PC6(4)
Points of chest and abdomen (EX-CA)	3	1	EX-CA1(3)
Triple energizer meridian of hand-shaoyang (TE)	2	2	TE5(1), TE14(1)
Points of upper extremities (EX-UE)	1	1	EX-UE1(1)
Points of lower extremities (EX-LE)	1	1	EX-LE3(1)

**Table 5:** Common acupoint group of acupuncture for primary osteoporosis.

No.	Acupoint group	Frequency	No.	Acupoint group	Frequency	No.	Acupoint group	Frequency
1	BL23, BL20	117	6	BL20, GV4	74	11	ST36, SP6	66
2	BL23, GV4	104	7	BL23, SP6	72	12	BL23, CV4	66
3	BL23, ST36	101	8	BL23, BL20, GV4	72	13	ST36, SP6, BL23	62
4	BL20, ST36	76	9	BL23, KI3	69	14	SP6, BL20	60
5	BL23, BL20, ST36	76	10	ST36, GV4	62	15	SP6, BL23, BL20	60

**Table 6:** Association rules of acupoints.

Nio	Acupoints	Confidence level/%	Number	Acupoints	Confidence level/%	Number	Acupoints	Confidence level/%
1	BL20, ST36 -> BL23	98.7	9	CV4 -> BL23	86.8	17	BL23, BL20 -> ST36	64.1
2	BL20 -> BL23	97.5	10	ST36, BL23 -> BL20	74.3	18	BL20 -> ST36	63.3
3	BL20, GV4 -> BL23	97.3	11	BL23, GV4 -> BL20	69.2	19	BL20 -> ST36, BL23	62.5
4	SP6 -> BL23	94.7	12	ST36 -> BL20	67.9	20	BL20 -> GV4	61.7
5	GV4 -> BL23	93.7	13	ST36 -> BL23, BL20	67.0	21	BL23, BL20 -> GV4	61.5
6	KI3 -> BL23	90.8	14	GV4 -> BL20	66.7	22	BL20 -> BL23, GV4	60.0
7	ST36 -> BL23	90.7	15	BL23 -> BL20	66.5			
8	SP6 -> ST36	86.8	16	GV4 -> BL23, BL20	64.9			

**Table 7:** Representative examples of major antiosteoporotic effects of core acupoints and potential mechanisms.

Acupoints	Beneficial Effects	Potential Mechanisms	Experimental Models Used	Ref. #
BL23	reduce the loss of bone mass, increase femoral BMD, improve the microstructure of bone tissue, promote bone formation, restore the amount of bone volume, improve bone architecture, enhance the secretion of testosterone	Upregulate the ratio of OPG/RANKL and LRP5, *β*-catenin and Runx2 expression; downregulate the expression of phosphorylated (p)-p38 and p-JNK	rodent models	[4, 35]
BL20	reduce the loss of bone mass, increase femoral BMD, improve the microstructure of bone tissue	Upregulate the ratio of OPG/RANKL and LRP5, *β*-catenin and Runx2 expression; downregulate the expression of phosphorylated (p)-p38 and p-JNK	rodent models	[4]
ST36	increase the tibia BMD, increased E_2_ levels	Reduced OPGL expression; upregulate the expression levels of RANKL mRNA	Rabbits, rodent models	[36, 37]
GV4	reduce the loss of bone mass, increase the femoral BMD, improve the microstructure of the bone tissue	Upregulate the ratio of OPG/RANKL expression; upregulate the expression levels of LRP5, *β*-catenin and Runx2	rodent models	[38]
SP6	increase BMD, increased E_2_ levels	Decrease the concentration of TNF*α* and IgM	Human body	[7]
CV4	increase E_2_ levels, promote the serum ALP level, compress the serum DPD, promote bone formation, decrease the bone absorb, increase the immune function, resist the apoptosis and delay the regressive changes in uterus, strengthen the homeostasis of body	Upregulate the expression levels of ER-*α* and ER-*α* mRNA in uterus, hypothalamus and spleen	rodent models	[39, 40]
KI3	increase BMD, E_2_ levels	Decrease the concentration of TNF*α* and IgM	Human body	[7]

MD = bone mineral density, JNK = c-Jun N-terminal kinase, OPGL = osteoprotegerin ligand, RANKL = receptor activator of NF-kB ligand, Ig = immunoglobulin, E_2_ = oestradiol, OPG = osteoprotegerin, TNF = tissue necrosis factor, ALP = alkaline phosphatase, DPD = deoxypyridinoline, ER-*α* = estrogen receptor-*α*, LRP = lipoprotein receptor-related protein, Runx = runt-related transcription factor.

## Data Availability

The detailed heterogeneity score, AMSTAR 2 score, and PRISMA score of included studies were shown in Additional Tables 1–3. The prescriptions were available from the corresponding author on reasonable request. The datasets or software generated and/or analyzed during the study were available in the Chinese Biomedical Medicine: http://www.sinomed.ac.cn/cross/; VIP Database: http://vip.hbsti.ac.cn/; Wanfang Data: http://www.wanfangdata.com.cn/index.html.

## Abbreviations

CBM: Chinese Biomedical Medicine
CNKI: China National Knowledge Infrastructure
AMSTAR: Assessment of Multiple Systematic Reviews tool
PRISMA: Preferred Reporting Items for Systematic Reviews and Meta-Analyse
RCT: Randomized control trial
CCT: Clinical control trial
BMD: Bone Mineral Density
BGP: Bone Gla protein
VAS: Visual analog scale
TCM: Traditional Chinese Medicine
E: Experimental group
C: Control group
OP: Osteoporosis
TCMISS: Traditional Chinese Medicine inheritance support system
AMSTAR: Assessment of Multiple Systematic Reviews
SD: Standard deviations
BL: Bladder meridian of foot-taiyang
GV: Governor vessel
CV: Conception vessel
GB: Gallbladder meridian of foot-shaoyang
ST: Stomach meridian of foot-yangming
SP: Spleen meridian of foot-taiyin
KI: Kidney meridian of foot-shaoyin
EX-B: Points of back
LR: Liver meridian of foot-jueyin
LI: Large intestine meridian of hand-yangming
LU: Lung meridian of hand-taiyin
SI: Small intestine meridian of hand-taiyang
PC: Pericardium meridian of hand-jueyin
EX-CA: Points of chest and abdomen
TE: Triple energizer meridian of hand-shaoyang
EX-UE: Points of upper extremities
EX-LE: Points of lower extremities
JNK: c-Jun N-terminal kinase
OPGL: Osteoprotegerin ligand
RANKL: Receptor activator of NF-kB ligand
Ig: Immunoglobulin
E2: Oestradiol
OPG: Osteoprotegerin
TNF: Tissue necrosis factor
ALP: Alkaline phosphatase
DPD: Deoxypyridinoline
ER-*α*: Estrogen receptor-*α*
LRP: Lipoprotein receptor-related protein
Runx: Runt-related transcription factor.

## Supplementary Materials

Supplementary Materials Additional Table 1: heterogeneity score of included studies. Additional Table 2: the Assessment of Multiple Systematic Reviews (AMSTAR) 2 tool. Additional Table 3: Preferred Reporting Items for Systematic Reviews and Meta-Analyses (PRISMA) 2009 Checklist.
