# Supplementary material for: Acupuncture for Primary Osteoporosis: Evidence, Potential Treatment Prescriptions, and Mechanisms
Source: Evid Based Complement Alternat Med. 2019 Jun 12;2019:2705263. doi: 10.1155/2019/2705263 (PMC6594290; doi:10.1155/2019/2705263)
Supplement: Supplementary Materials — Additional Table 1: heterogeneity score of included studies. Additional Table 2: the Assessment of Multiple Systematic Reviews (AMSTAR) 2 tool. Additional Table 3: Preferred Reporting Items for Systematic Reviews and Meta-Analyses (PRISMA) 2009 Checklist. [file 2705263.f1.doc]

**Supplementary Table 1 to 3**

| **Additional Table 1. Heterogeneity score of included studies.** | | | | |
| --- | --- | --- | --- | --- |
| Studies | Studies’ results (number)^*^ | | | Heterogeneity score^#^ |
|  | Low heterogeneity | Moderate heterogeneity | Significant heterogeneity |  |
| Hong Pan et al. 2018 [26] | 2 | 3 | 6 | 1.64 |
| Jinfeng Chen et al. 2018 [27] | 0 | 2 | 3 | 1.4 |
| Ding Luo et al. 2018 [5] | 3 | 0 | 2 | 2.2 |
| Xiaotong Wang et al. 2018 [28] | 5 | 0 | 0 | 3 |
| Ding Luo et al. 2017 [29] | 3 | 0 | 5 | 1.75 |
| Yanan Wu et al. 2017 [30] | 1 | 0 | 3 | 1.5 |
| Li Xiao et al. 2016 [31] | 3 | 0 | 0 | 3 |
| Longyun Zhou et al. 2016 [32] | 2 | 0 | 2 | 2 |
| Sheng Li et al. 2014 [33] | 3 | 0 | 0 | 3 |
| Juntao Xu et al. 2014 [34] | 2 | 0 | 1 | 2.33 |
| **Note**: *Statistical heterogeneity was assessed using the Chi-squared test and I^2^ values*. I^2^*＞75% indicated significant heterogeneity, 50%≤*I^2^*≤75% was regarded as moderate heterogeneity, and 0%≤*I^2^*≤50% was defined as indicating low heterogeneity. [*From: Higgins JP, Thompson SG, Deeks JJ, Altman DG. Measuring inconsistency in meta-analyses. BMJ. 2003;327(7414):557–560*. doi: 10.1136/bmj.327.7414.557]  # Heterogeneity score=(Low*3+Moderate*2+Significant*1)/(Low+Moderate+Significant) | | | | |

| **Additional Table 2. The Assessment of Multiple Systematic Reviews (AMSTAR) 2 tool.** | | | |
| --- | --- | --- | --- |
| **Checklist item** | **N** | **Detailed description** | **Studies (Score)^*^** |
| 1. Did the research questions and inclusion criteria for the review include the components of PICO? | 1 | • For Yes:  □Population  □Intervention  □Comparator group  □Outcome  • Optional (recommended)  □Timeframe for follow-up | Ding Luo et al. 2018 (1), Hong Pan et al. 2018 (1), Jinfeng Chen et al. 2018 (1), Sheng Li et al. 2014 (1), Ding Luo et al. 2017 (1), Xiaotong Wang et al. 2018 (1), Yanan Wu et al. 2017 (1), Li Xiao et al. 2016 (1), Juntao Xu et al. 2014 (1), Longyun Zhou et al. 2016 (1) |
| 1. Did the report of the review contain an explicit statement that the review methods were established prior to the conduct of the review and did the report justify any significant deviations from the protocol? | 2 | • For Partial Yes:  The authors state that they had a written protocol or guide that included ALL the following:  □review question(s)  □a search strategy  □inclusion/exclusion criteria  □a risk of bias assessment  • For Yes:  As for partial yes, plus the protocol should be registered and should also have specified:  □a meta-analysis/synthesis plan, if appropriate, and  □a plan for investigating causes of heterogeneity  □justification for any deviations from the protocol | Ding Luo et al. 2018 (1), Hong Pan et al. 2018 (0), Jinfeng Chen et al. 2018 (0), Sheng Li et al. 2014 (0), Ding Luo et al. 2017 (0), Xiaotong Wang et al. 2018 (0), Yanan Wu et al. 2017 (0), Li Xiao et al. 2016 (0), Juntao Xu et al. 2014 (0), Longyun Zhou et al. 2016 (0) |
| 1. Did the review authors explain their selection of the study designs for inclusion in the review? | 3 | • For Yes, the review should satisfy ONE of the following:  □Explanation for including only RCTs  □OR Explanation for including only NRSI  □OR Explanation for including both RCTs and NRSI | Ding Luo et al. 2018 (1), Hong Pan et al. 2018 (1), Jinfeng Chen et al. 2018 (1), Sheng Li et al. 2014 (1), Ding Luo et al. 2017 (1), Xiaotong Wang et al. 2018 (1), Yanan Wu et al. 2017 (1), Li Xiao et al. 2016 (1), Juntao Xu et al. 2014 (1), Longyun Zhou et al. 2016 (1) |
| 1. Did the review authors use a comprehensive literature search strategy? | 4 | • For Partial Yes (all the following):  □searched at least 2 databases (relevant to research question)  □provided key word and/or search strategy  □justified publication restrictions (eg, language)  • For Yes, should also have (all the following):  □searched the reference lists/bibliographies of included studies  □searched trial/study registries  □included/consulted content experts in the field  □where relevant, searched for grey literature  □conducted search within 24 months of completion of the review | Ding Luo et al. 2018 (1), Hong Pan et al. 2018 (1), Jinfeng Chen et al. 2018 (0.5), Sheng Li et al. 2014 (0.5), Ding Luo et al. 2017 (0.5), Xiaotong Wang et al. 2018 (0.5), Yanan Wu et al. 2017 (1), Li Xiao et al. 2016 (1), Juntao Xu et al. 2014 (0.5), Longyun Zhou et al. 2016 (1) |
| 1. Did the review authors perform study selection in duplicate? | 5 | • For Yes, either ONE of the following:  □at least two reviewers independently agreed on selection of eligible studies and achieved consensus on which studies to include  □OR two reviewers selected a sample of eligible studies and achieved good agreement (at least 80 per cent), with the remainder selected by one reviewer | Ding Luo et al. 2018 (1), Hong Pan et al. 2018 (1), Jinfeng Chen et al. 2018 (0), Sheng Li et al. 2014 (0), Ding Luo et al. 2017 (0), Xiaotong Wang et al. 2018 (1), Yanan Wu et al. 2017 (1), Li Xiao et al. 2016 (1), Juntao Xu et al. 2014 (0), Longyun Zhou et al. 2016 (1) |
| 1. Did the review authors perform data extraction in duplicate? | 6 | • For Yes, either ONE of the following:  □at least two reviewers achieved consensus on which data to extract from included studies  □OR two reviewers extracted data from a sample of eligible studies and achieved good agreement (at least 80 per cent), with the remainder extracted by one reviewer | Ding Luo et al. 2018 (1), Hong Pan et al. 2018 (1), Jinfeng Chen et al. 2018 (1), Sheng Li et al. 2014 (1), Ding Luo et al. 2017 (1), Xiaotong Wang et al. 2018 (1), Yanan Wu et al. 2017 (1), Li Xiao et al. 2016 (1), Juntao Xu et al. 2014 (1), Longyun Zhou et al. 2016 (1) |
| 1. Did the review authors provide a list of excluded studies and justify the exclusions? | 7 | • For Partial Yes:  □provided a list of all potentially relevant studies that were read in full text form but excluded from the review  • For Yes, must also have:  □Justified the exclusion from the review of each potentially relevant study | Ding Luo et al. 2018 (0), Hong Pan et al. 2018 (0), Jinfeng Chen et al. 2018 (0), Sheng Li et al. 2014 (0), Ding Luo et al. 2017 (0), Xiaotong Wang et al. 2018 (0), Yanan Wu et al. 2017 (0), Li Xiao et al. 2016 (0), Juntao Xu et al. 2014 (0), Longyun Zhou et al. 2016 (0) |
| 1. Did the review authors describe the included studies in adequate detail? | 8 | • For Partial Yes (ALL the following):  □described populations  □described interventions  □described comparators  □described outcomes  □described research designs  • For Yes, should also have ALL the following:  □described population in detail  □described intervention and comparator in detail (including doses where relevant)  □described study’s setting  □timeframe for follow-up | Ding Luo et al. 2018 (0.5), Hong Pan et al. 2018 (1), Jinfeng Chen et al. 2018 (0.5), Sheng Li et al. 2014 (1), Ding Luo et al. 2017 (0.5), Xiaotong Wang et al. 2018 (0.5), Yanan Wu et al. 2017 (0.5), Li Xiao et al. 2016 (0.5), Juntao Xu et al. 2014 (0.5), Longyun Zhou et al. 2016 (0.5) |
| 1. individual studies that were included in the review? | 9 | **RCTs**  • For Partial Yes, must have assessed risk of bias (RoB) from  □unconcealed allocation, and  □lack of blinding of patients and assessors when assessing outcomes (unnecessary for objective outcomes such as all cause mortality)  • For Yes, must also have assessed RoB from:  □allocation sequence that was not truly random, and  □selection of the reported result from among multiple measurements or analyses of a specified outcome  **NRSI**  • For Partial Yes, must have assessed RoB:  □from confounding, and  □from selection bias  • For Yes, must also have assessed RoB:  □methods used to ascertain exposures and outcomes, and  □selection of the reported result from among multiple measurements or analyses of a specified outcome | Ding Luo et al. 2018 (1), Hong Pan et al. 2018 (1), Jinfeng Chen et al. 2018 (1), Sheng Li et al. 2014 (1), Ding Luo et al. 2017 (1), Xiaotong Wang et al. 2018 (1), Yanan Wu et al. 2017 (1), Li Xiao et al. 2016 (1), Juntao Xu et al. 2014 (1), Longyun Zhou et al. 2016 (1) |
| 1. Did the review authors report on the sources of funding for the studies included in the review? | 10 | • For Yes  □Must have reported on the sources of funding for individual studies included in the review. Note: Reporting that the reviewers looked for this information but it was not reported by study authors also qualifies | Ding Luo et al. 2018 (0), Hong Pan et al. 2018 (0), Jinfeng Chen et al. 2018 (0), Sheng Li et al. 2014 (0), Ding Luo et al. 2017 (0), Xiaotong Wang et al. 2018 (0), Yanan Wu et al. 2017 (0), Li Xiao et al. 2016 (0), Juntao Xu et al. 2014 (0), Longyun Zhou et al. 2016 (0) |
| 1. If meta-analysis was performed did the review authors use appropriate methods for statistical combination of results? | 11 | **RCTs**  • For Yes:  □The authors justified combining the data in a meta-analysis  □AND they used an appropriate weighted technique to combine study results and adjusted for heterogeneity if present  □AND investigated the causes of any heterogeneity  **For NRSI**  • For Yes:  □The authors justified combining the data in a meta-analysis  □AND they used an appropriate weighted technique to combine study results, adjusting for heterogeneity if present  □AND they statistically combined effect estimates from NRSI that were adjusted for confounding, rather than combining raw data, or justified combining raw data when adjusted effect estimates were not available  □AND they reported separate summary estimates for RCTs and NRSI separately when both were included in the review | Ding Luo et al. 2018 (1), Hong Pan et al. 2018 (1), Jinfeng Chen et al. 2018 (1), Sheng Li et al. 2014 (1), Ding Luo et al. 2017 (1), Xiaotong Wang et al. 2018 (1), Yanan Wu et al. 2017 (1), Li Xiao et al. 2016 (1), Juntao Xu et al. 2014 (1), Longyun Zhou et al. 2016 (1) |
| 1. If meta-analysis was performed, did the review authors assess the potential impact of RoB in individual studies on the results of the meta-analysis or other evidence synthesis? | 12 | • For Yes:  □included only low risk of bias RCTs  □OR, if the pooled estimate was based on RCTs and/or NRSI at variable RoB, the authors performed analyses to investigate possible impact of RoB on summary estimates of effect | Ding Luo et al. 2018 (0), Hong Pan et al. 2018 (0), Jinfeng Chen et al. 2018 (0), Sheng Li et al. 2014 (0), Ding Luo et al. 2017 (0), Xiaotong Wang et al. 2018 (0), Yanan Wu et al. 2017 (0), Li Xiao et al. 2016 (0), Juntao Xu et al. 2014 (0), Longyun Zhou et al. 2016 (0) |
| 1. Did the review authors account for RoB in individual studies when interpreting/discussing the results of the review? | 13 | • For Yes:  □included only low risk of bias RCTs  □OR, if RCTs with moderate or high RoB, or NRSI were included the review provided a discussion of the likely impact of RoB on the results | Ding Luo et al. 2018 (1), Hong Pan et al. 2018 (1), Jinfeng Chen et al. 2018 (1), Sheng Li et al. 2014 (1), Ding Luo et al. 2017 (1), Xiaotong Wang et al. 2018 (1), Yanan Wu et al. 2017 (1), Li Xiao et al. 2016 (1), Juntao Xu et al. 2014 (1), Longyun Zhou et al. 2016 (1) |
| 1. Did the review authors provide a satisfactory explanation for, and discussion of, any heterogeneity observed in the results of the review? | 14 | • For Yes:  □There was no significant heterogeneity in the results  □OR if heterogeneity was present the authors performed an investigation of sources of any heterogeneity in the results and discussed the impact of this on the results of the review | Ding Luo et al. 2018 (1), Hong Pan et al. 2018 (1), Jinfeng Chen et al. 2018 (1), Sheng Li et al. 2014 (0), Ding Luo et al. 2017 (0), Xiaotong Wang et al. 2018 (1), Yanan Wu et al. 2017 (0), Li Xiao et al. 2016 (1), Juntao Xu et al. 2014 (0), Longyun Zhou et al. 2016 (1) |
| 1. If they performed quantitative synthesis did the review authors carry out an adequate investigation of publication bias (small study bias) and discuss its likely impact on the results of the review? | 15 | • For Yes:  □performed graphical or statistical tests for publication bias and discussed the likelihood and magnitude of impact of publication bias | Ding Luo et al. 2018 (0), Hong Pan et al. 2018 (1), Jinfeng Chen et al. 2018 (1), Sheng Li et al. 2014 (0), Ding Luo et al. 2017 (1), Xiaotong Wang et al. 2018 (0), Yanan Wu et al. 2017 (1), Li Xiao et al. 2016 (1), Juntao Xu et al. 2014 (0), Longyun Zhou et al. 2016 (0) |
| 1. Did the review authors report any potential sources of conflict of interest, including any funding they received for conducting the review? | 16 | • For Yes:  □The authors reported no competing interests OR  □The authors described their funding sources and how they managed potential conflicts of interest | Ding Luo et al. 2018 (1), Hong Pan et al. 2018 (0), Jinfeng Chen et al. 2018 (0), Sheng Li et al. 2014 (0), Ding Luo et al. 2017 (0), Xiaotong Wang et al. 2018 (0), Yanan Wu et al. 2017 (0), Li Xiao et al. 2016 (0), Juntao Xu et al. 2014 (0), Longyun Zhou et al. 2016 (0) |
| **Note**: *If a study fit this item, it will be registered here. 1point: Yes; 0.5 points: Partial Yes; 0 point: No.  *From: Shea BJ, Reeves BC, Wells G, et al. AMSTAR 2: a critical appraisal tool for systematic reviews that include randomised or non-randomised studies of healthcare interventions, or both. BMJ 2017; 358: j4008. doi: 10.1136/bmj.j4008.* | | | |

| **Additional Table 3. Preferred Reporting Items for Systematic Reviews and Meta-Analyses (PRISMA) 2009 Checklist.** | | | |
| --- | --- | --- | --- |
| **Section/topic** | **N** | **Checklist item** | **Studies (Score)^*^** |
| **TITLE** | | | |
| Title | 1 | Identify the report as a systematic review, meta-analysis, or both. | Ding Luo et al. 2018 (2), Hong Pan et al. 2018 (2), Jinfeng Chen et al. 2018 (2), Sheng Li et al. 2014 (2), Ding Luo et al. 2017 (2), Xiaotong Wang et al. 2018 (2), Yaanan Wu et al. 2017 (2), Li Xiao et al. 2016 (2), Juntao Xu et al. 2014 (2), Longyun Zhou et al. 2016 (2) |
| **ABSTRACT** | | |  |
| Structured summary | 2 | Provide a structured summary including, as applicable: background; objectives; data sources; study eligibility criteria, participants, and interventions; study appraisal and synthesis methods; results; limitations; conclusions and implications of key findings; systematic review registration number. | Ding Luo et al. 2018 (2), Hong Pan et al. 2018 (1), Jinfeng Chen et al. 2018 (1), Sheng Li et al. 2014 (1), Ding Luo et al. 2017 (1), Xiaotong Wang et al. 2018 (1), Yaanan Wu et al. 2017 (1), Li Xiao et al. 2016 (1), Juntao Xu et al. 2014 (1), Longyun Zhou et al. 2016 (1) |
| **INTRODUCTION** | | | |
| Rationale | 3 | Describe the rationale for the review in the context of what is already known. | Ding Luo et al. 2018 (2), Hong Pan et al. 2018 (2), Jinfeng Chen et al. 2018 (2), Sheng Li et al. 2014 (2), Ding Luo et al. 2017 (2), Xiaotong Wang et al. 2018 (2), Yaanan Wu et al. 2017 (2), Li Xiao et al. 2016 (2), Juntao Xu et al. 2014 (2), Longyun Zhou et al. 2016 (2) |
| Objectives | 4 | Provide an explicit statement of questions being addressed with reference to participants, interventions, comparisons, outcomes, and study design (PICOS). | Ding Luo et al. 2018 (2), Hong Pan et al. 2018 (2), Jinfeng Chen et al. 2018 (2), Sheng Li et al. 2014 (2), Ding Luo et al. 2017 (2), Xiaotong Wang et al. 2018 (2), Yaanan Wu et al. 2017 (2), Li Xiao et al. 2016 (2), Juntao Xu et al. 2014 (2), Longyun Zhou et al. 2016 (2) |
| **METHODS** | | | |
| Protocol and registration | 5 | Indicate if a review protocol exists, if and where it can be accessed (e.g., Web address), and, if available, provide registration information including registration number. | Ding Luo et al. 2018 (2), Hong Pan et al. 2018 (0), Jinfeng Chen et al. 2018 (0), Sheng Li et al. 2014 (0), Ding Luo et al. 2017 (0), Xiaotong Wang et al. 2018 (0), Yaanan Wu et al. 2017 (0), Li Xiao et al. 2016 (0), Juntao Xu et al. 2014 (0), Longyun Zhou et al. 2016 (0) |
| Eligibility criteria | 6 | Specify study characteristics (e.g., PICOS, length of follow-up) and report characteristics (e.g., years considered, language, publication status) used as criteria for eligibility, giving rationale. | Ding Luo et al. 2018 (2), Hong Pan et al. 2018 (2), Jinfeng Chen et al. 2018 (2), Sheng Li et al. 2014 (1), Ding Luo et al. 2017 (1), Xiaotong Wang et al. 2018 (1), Yaanan Wu et al. 2017 (1), Li Xiao et al. 2016 (1), Juntao Xu et al. 2014 (1), Longyun Zhou et al. 2016 (2) |
| Information sources | 7 | Describe all information sources (e.g., databases with dates of coverage, contact with study authors to identify additional studies) in the search and date last searched. | Ding Luo et al. 2018 (1), Hong Pan et al. 2018 (1), Jinfeng Chen et al. 2018 (2), Sheng Li et al. 2014 (1), Ding Luo et al. 2017 (1), Xiaotong Wang et al. 2018 (1), Yaanan Wu et al. 2017 (1), Li Xiao et al. 2016 (1), Juntao Xu et al. 2014 (1), Longyun Zhou et al. 2016 (1) |
| Search | 8 | Present full electronic search strategy for at least one database, including any limits used, such that it could be repeated. | Ding Luo et al. 2018 (2), Hong Pan et al. 2018 (2), Jinfeng Chen et al. 2018 (1), Sheng Li et al. 2014 (1), Ding Luo et al. 2017 (1), Xiaotong Wang et al. 2018 (2), Yaanan Wu et al. 2017 (1), Li Xiao et al. 2016 (1), Juntao Xu et al. 2014 (0), Longyun Zhou et al. 2016 (1) |
| Study selection | 9 | State the process for selecting studies (i.e., screening, eligibility, included in systematic review, and, if applicable, included in the meta-analysis). | Ding Luo et al. 2018 (2), Hong Pan et al. 2018 (2), Jinfeng Chen et al. 2018 (2), Sheng Li et al. 2014 (2), Ding Luo et al. 2017 (2), Xiaotong Wang et al. 2018 (2), Yaanan Wu et al. 2017 (2), Li Xiao et al. 2016 (2), Juntao Xu et al. 2014 (1), Longyun Zhou et al. 2016 (2) |
| Data collection process | 10 | Describe method of data extraction from reports (e.g., piloted forms, independently, in duplicate) and any processes for obtaining and confirming data from investigators. | Ding Luo et al. 2018 (2), Hong Pan et al. 2018 (2), Jinfeng Chen et al. 2018 (2), Sheng Li et al. 2014 (2), Ding Luo et al. 2017 (2), Xiaotong Wang et al. 2018 (2), Yaanan Wu et al. 2017 (2), Li Xiao et al. 2016 (2), Juntao Xu et al. 2014 (2), Longyun Zhou et al. 2016 (2) |
| Data items | 11 | List and define all variables for which data were sought (e.g., PICOS, funding sources) and any assumptions and simplifications made. | Ding Luo et al. 2018 (1), Hong Pan et al. 2018 (1), Jinfeng Chen et al. 2018 (1), Sheng Li et al. 2014 (1), Ding Luo et al. 2017 (1), Xiaotong Wang et al. 2018 (1), Yaanan Wu et al. 2017 (1), Li Xiao et al. 2016 (1), Juntao Xu et al. 2014 (1), Longyun Zhou et al. 2016 (1) |
| Risk of bias in individual studies | 12 | Describe methods used for assessing risk of bias of individual studies (including specification of whether this was done at the study or outcome level), and how this information is to be used in any data synthesis. | Ding Luo et al. 2018 (2), Hong Pan et al. 2018 (2), Jinfeng Chen et al. 2018 (2), Sheng Li et al. 2014 (2), Ding Luo et al. 2017 (2), Xiaotong Wang et al. 2018 (2), Yaanan Wu et al. 2017 (2), Li Xiao et al. 2016 (1), Juntao Xu et al. 2014 (1), Longyun Zhou et al. 2016 (1) |
| Summary measures | 13 | State the principal summary measures (e.g., risk ratio, difference in means). | Ding Luo et al. 2018 (2), Hong Pan et al. 2018 (2), Jinfeng Chen et al. 2018 (2), Sheng Li et al. 2014 (2), Ding Luo et al. 2017 (2), Xiaotong Wang et al. 2018 (2), Yaanan Wu et al. 2017 (2), Li Xiao et al. 2016 (2), Juntao Xu et al. 2014 (2), Longyun Zhou et al. 2016 (2) |
| Synthesis of results | 14 | Describe the methods of handling data and combining results of studies, if done, including measures of consistency (e.g., I^2^) for each meta-analysis. | Ding Luo et al. 2018 (2), Hong Pan et al. 2018 (2), Jinfeng Chen et al. 2018 (2), Sheng Li et al. 2014 (0), Ding Luo et al. 2017 (2), Xiaotong Wang et al. 2018 (2), Yaanan Wu et al. 2017 (2), Li Xiao et al. 2016 (2), Juntao Xu et al. 2014 (2), Longyun Zhou et al. 2016 (2) |
| Risk of bias across studies | 15 | Specify any assessment of risk of bias that may affect the cumulative evidence (e.g., publication bias, selective reporting within studies). | Ding Luo et al. 2018 (0), Hong Pan et al. 2018 (2), Jinfeng Chen et al. 2018 (2), Sheng Li et al. 2014 (0), Ding Luo et al. 2017 (2), Xiaotong Wang et al. 2018 (1), Yaanan Wu et al. 2017 (2), Li Xiao et al. 2016 (1), Juntao Xu et al. 2014 (0), Longyun Zhou et al. 2016 (2) |
| Additional analyses | 16 | Describe methods of additional analyses (e.g., sensitivity or subgroup analyses, meta-regression), if done, indicating which were pre-specified. | Ding Luo et al. 2018 (2), Hong Pan et al. 2018 (2), Jinfeng Chen et al. 2018 (2), Sheng Li et al. 2014 (0), Ding Luo et al. 2017 (2), Xiaotong Wang et al. 2018 (2), Yaanan Wu et al. 2017 (2), Li Xiao et al. 2016 (2), Juntao Xu et al. 2014 (0), Longyun Zhou et al. 2016 (2) |
| **RESULTS** | | | |
| Study selection | 17 | Give numbers of studies screened, assessed for eligibility, and included in the review, with reasons for exclusions at each stage, ideally with a flow diagram. | Ding Luo et al. 2018 (2), Hong Pan et al. 2018 (2), Jinfeng Chen et al. 2018 (2), Sheng Li et al. 2014 (2), Ding Luo et al. 2017 (2), Xiaotong Wang et al. 2018 (2), Yaanan Wu et al. 2017 (0), Li Xiao et al. 2016 (2), Juntao Xu et al. 2014 (1), Longyun Zhou et al. 2016 (2) |
| Study characteristics | 18 | For each study, present characteristics for which data were extracted (e.g., study size, PICOS, follow-up period) and provide the citations. | Ding Luo et al. 2018 (2), Hong Pan et al. 2018 (2), Jinfeng Chen et al. 2018 (2), Sheng Li et al. 2014 (2), Ding Luo et al. 2017 (2), Xiaotong Wang et al. 2018 (2), Yaanan Wu et al. 2017 (2), Li Xiao et al. 2016 (2), Juntao Xu et al. 2014 (1), Longyun Zhou et al. 2016 (2) |
| Risk of bias within studies | 19 | Present data on risk of bias of each study and, if available, any outcome level assessment (see item 12). | Ding Luo et al. 2018 (2), Hong Pan et al. 2018 (2), Jinfeng Chen et al. 2018 (2), Sheng Li et al. 2014 (2), Ding Luo et al. 2017 (2), Xiaotong Wang et al. 2018 (2), Yaanan Wu et al. 2017 (2), Li Xiao et al. 2016 (1), Juntao Xu et al. 2014 (0), Longyun Zhou et al. 2016 (1) |
| Results of individual studies | 20 | For all outcomes considered (benefits or harms), present, for each study: (a) simple summary data for each intervention group (b) effect estimates and confidence intervals, ideally with a forest plot. | Ding Luo et al. 2018 (2), Hong Pan et al. 2018 (2), Jinfeng Chen et al. 2018 (2), Sheng Li et al. 2014 (2), Ding Luo et al. 2017 (2), Xiaotong Wang et al. 2018 (2), Yaanan Wu et al. 2017 (2), Li Xiao et al. 2016 (2), Juntao Xu et al. 2014 (2), Longyun Zhou et al. 2016 (2) |
| Synthesis of results | 21 | Present results of each meta-analysis done, including confidence intervals and measures of consistency. | Ding Luo et al. 2018 (2), Hong Pan et al. 2018 (2), Jinfeng Chen et al. 2018 (2), Sheng Li et al. 2014 (2), Ding Luo et al. 2017 (1), Xiaotong Wang et al. 2018 (2), Yaanan Wu et al. 2017 (2), Li Xiao et al. 2016 (2), Longyun Zhou et al. 2016 (2) |
| Risk of bias across studies | 22 | Present results of any assessment of risk of bias across studies (see Item 15). | Ding Luo et al. 2018 (2), Hong Pan et al. 2018 (2), Jinfeng Chen et al. 2018 (0), Sheng Li et al. 2014 (0), Ding Luo et al. 2017 (0), Xiaotong Wang et al. 2018 (0), Yaanan Wu et al. 2017 (2), Li Xiao et al. 2016 (1), Juntao Xu et al. 2014 (0), Longyun Zhou et al. 2016 (2) |
| Additional analysis | 23 | Give results of additional analyses, if done (e.g., sensitivity or subgroup analyses, meta-regression [see Item 16]). | Ding Luo et al. 2018 (1), Hong Pan et al. 2018 (2), Jinfeng Chen et al. 2018 (0), Sheng Li et al. 2014 (0), Ding Luo et al. 2017 (0), Xiaotong Wang et al. 2018 (2), Yaanan Wu et al. 2017 (2), Li Xiao et al. 2016 (2), Juntao Xu et al. 2014 (0), Longyun Zhou et al. 2016 (2) |
| **DISCUSSION** | | | |
| Summary of evidence | 24 | Summarize the main findings including the strength of evidence for each main outcome; consider their relevance to key groups (e.g., healthcare providers, users, and policy makers). | Ding Luo et al. 2018 (1), Hong Pan et al. 2018 (2), Jinfeng Chen et al. 2018 (0), Sheng Li et al. 2014 (1), Ding Luo et al. 2017 (0), Xiaotong Wang et al. 2018 (1), Yaanan Wu et al. 2017 (1), Li Xiao et al. 2016 (1), Juntao Xu et al. 2014 (0), Longyun Zhou et al. 2016 (1) |
| Limitations | 25 | Discuss limitations at study and outcome level (e.g., risk of bias), and at review-level (e.g., incomplete retrieval of identified research, reporting bias). | Ding Luo et al. 2018 (1), Hong Pan et al. 2018 (2), Jinfeng Chen et al. 2018 (2), Sheng Li et al. 2014 (2), Ding Luo et al. 2017 (1), Xiaotong Wang et al. 2018 (1), Yaanan Wu et al. 2017 (1), Li Xiao et al. 2016 (2), Juntao Xu et al. 2014 (2), Longyun Zhou et al. 2016 (2) |
| Conclusions | 26 | Provide a general interpretation of the results in the context of other evidence, and implications for future research. | Ding Luo et al. 2018 (2), Hong Pan et al. 2018 (2), Jinfeng Chen et al. 2018 (2), Sheng Li et al. 2014 (2), Ding Luo et al. 2017 (0), Xiaotong Wang et al. 2018 (2), Yaanan Wu et al. 2017 (2), Li Xiao et al. 2016 (2), Juntao Xu et al. 2014 (1), Longyun Zhou et al. 2016 (2) |
| **FUNDING** | | | |
| Funding | 27 | Describe sources of funding for the systematic review and other support (e.g., supply of data); role of funders for the systematic review. | Ding Luo et al. 2018 (1), Hong Pan et al. 2018 (0), Jinfeng Chen et al. 2018 (0), Sheng Li et al. 2014 (1), Ding Luo et al. 2017 (1), Xiaotong Wang et al. 2018 (1), Yaanan Wu et al. 2017 (0), Li Xiao et al. 2016 (1), Juntao Xu et al. 2014 (0), Longyun Zhou et al. 2016 (1) |
| **Note**:* 2 points: Exactly matches this item; 1 point: Partially matches this item; 0 point: not matches this item.  *From: Moher D, Liberati A, Tetzlaff J, Altman DG, The PRISMA Group. Preferred Reporting Items for Systematic Reviews and Meta-Analyses: The PRISMA Statement. PLoS Med. 2009;6(7): e1000097. doi:10.1371/journal.pmed1000097* | | | |
